# Supplementary material for: Momentum-resolved visualization of electronic evolution in doping a Mott insulator
Source: Nat Commun. 2021 Mar 1;12:1356. doi: 10.1038/s41467-021-21605-6 (PMC7921433; doi:10.1038/s41467-021-21605-6)
Supplement: Supplementary file 1 — Supplementary Information [file 41467_2021_21605_MOESM1_ESM.pdf]

Supplementary Materials for

Momentum-Resolved Visualization of Electronic Evolution in Doping a Mott Insulator

Cheng Hu[1,2†‡], Jianfa Zhao[1,2†], Qiang Gao[1,2], Hongtao Yan[1,2], Hongtao Rong[1,2], Jianwei Huang[1,2], Jing Liu[1,2,3], Yongqing Cai[1,2], Cong Li[1,2], Hao Chen[1,2], Lin Zhao[1], Guodong Liu[1,2,4], Changqing Jin[1,2,4*], Zuyan Xu[5], Tao Xiang[1,2,3,4] and X. J. Zhou[1,2,3,4*]

[1]*National Lab for Superconductivity,*

Beijing National laboratory for Condensed Matter Physics,

Institute of Physics, Chinese Academy of Sciences, Beijing 100190, China

[2]*University of Chinese Academy of Sciences, Beijing 100049, China*

[3]*Beijing Academy of Quantum Information Sciences, Beijing 100193, China*

[4]*Songshan Lake Materials Laboratory, Dongguan 523808, China*

[5]*Technical Institute of Physics and Chemistry,*

Chinese Academy of Sciences, Beijing 100190, China

[†]*These authors contribute equally to the present work.*

[‡]*Present address: Department of Materials Science and Engineering,*

Cornell University, Ithaca, New York 14853, USA

[*]*Corresponding author: XJZhou@iphy.ac.cn and Jin@iphy.ac.cn*

(Dated: January 26, 2021)

Supplementary Note 1. Sample charging effect test

In the photoemission process, electrons in the measured sample are kicked out by the incident light. The escaped electrons are compensated by electrons flowing into the sample from the grounding, keeping the sample constantly in an electrically neutral condition. This compensation process can be readily realized in good conducting materials. However, when the sample is a poor conductor or an insulator, the electrons flowing from the grounding to the sample are blocked and may not be sufficient to compensate the lost electrons in the photoemission process, causing an accumulation of positive charges on the sample. The positive charges will attract the out-going photoelectrons, reducing their kinetic energy that corresponds to an energy shift to higher binding energy in the measured photoemission spectrum. Such a charging effect depends on the conducting property of the sample and the incident photon flux. Poor conductivity of the sample and large photon flux will result in a more serious charging effect. To carry out normal photoemission measurement and get intrinsic signal from a less-conductive sample, one should try to avoid the sample charging problem by reducing the photon flux, or maintaining a relatively high conductivity by measuring the sample at high temperature.

The $Ca_3Cu_2O_4Cl_2$ (CCOC326) sample is a perfect insulator which makes the photoemission measurements challenging. To avoid the sample charging effect, it is preferable to measure the sample at high temperature. On the other hand, for our present Rb-deposition measurements, in order to have sufficient Rb absorption on the sample surface to introduce electron doping over a relatively wide range, the sample needs to be measured at lower tem-

perature. To compromise between the charging effect and the Rb deposition efficiency, we tested the sample charging effect at different photon flux and at different temperatures, as shown in Supplementary Fig. 1. We find that the charging effect is negligible when the sample is measured at 50 K and above (Supplementary Fig. 1b). Then we chose the sample temperature around 50 K during our measurements. We note that, since CCOC326 is more conductive than its single-layer counterpart $Ca_2CuO_2Cl_2$ (CCOC214), this makes it possible to measure CCOC326 at much lower temperature than that for CCOC214. This is critical to the success of the experiment to realize sufficient and stable Rb absorption on the CCOC326 sample surface for electron doping. During the measurements, we frequently varied the photon flux to make sure that little charging effect is present in all the measurements.

Supplementary Note 2. Electronic structure of pristine $Ca_3Cu_2O_4Cl_2$

Supplementary Fig. 2 shows the measured electronic structure of pristine $Ca_3Cu_2O_4Cl_2$, including the constant energy contours (Supplementary Fig. 2a), band structure along high symmetry momentum cuts (Supplementary Fig. 2b and Supplementary Fig. 2d), the corresponding photoemission spectra (EDCs) (Supplementary Fig. 2c and Supplementary Fig. 2e), and two EDCs at the ($\pi/2$,$\pi/2$) nodal point and the (-π,0) antinodal point (Supplementary Fig. 2f). The overall electronic structure is similar to its single-layer counterpart $Ca_2CuO_2Cl_2$[1] except for slightly smaller energy gap and sharper spectral features observed in CCOC326. There is no spectral weight at the Fermi level (Supplementary Fig. 2a), consistent with its insulating nature. The top of the charge transfer band along the nodal direction (Supplemen-

tary Fig. 2b and Supplementary Fig. 2c) lies at ∼0.3 eV below the Fermi level. The charge transfer band near (-π,0) is much weaker in its intensity than that at the ($\pi/2$,$\pi/2$) nodal point and its EDC peak position lies at a higher binding energy, as shown in Supplementary Fig. 2f.

Supplementary Note 3. Raw data of band structure evolution with Rb deposition in $Ca_3Cu_2O_4Cl_2$

Supplementary Fig. 3 shows raw data of the band structure measured along three momentum cuts at different Rb deposition stages. Here the energy scale is the electron kinetic energy obtained directly from the measured data. In the photoemission process, the photoelectron kinetic energy does not represent an intrinsic quantity because it relies on the work function of the ARPES instrument and the used photon energy. The energy is usually referenced to the Fermi level (E_F) which is obtained by measuring on the Fermi cutoff of a gold foil. When the sample is electrically connected to the gold foil, the sample and the gold are expected to have the same Fermi level.

For the pristine CCOC326, as usual, we obtained the Fermi level from measuring the gold Fermi cutoff and mark the Fermi level position on the measured data of CCOC326, as shown by the white dashed lines in the three leftmost panels in Supplementary Fig. 3. The observed bands are below the Fermi level. The measurements are repeated on many CCOC326 samples and the results are reproducible and stable against the photon flux and the measurement time.

As soon as we deposit Rb on the CCOC326 sample surface, we started to see spectral weight at higher kinetic energy above the initially observed bands

in pristine CCOC326. These newly-developed spectral weight gets stronger with increasing Rb deposition, as seen in Supplementary Fig. 3. Surprisingly, the new states lie above the Fermi level position that is determined for the pristine CCOC326 from the usual gold Fermi cutoff measurement. Such Rb deposition process were repeated a couple of times, the measured results are highly reproducible. We note that, after each Rb deposition stage, the measured data along three momentum cuts are stable against the photon flux and measurement time. Little sample charging effect was involved in all the measurements.

The measured data show clear momentum dependence; they behave quite differently along the three momentum cuts. They also exhibit systematic variation with Rb deposition. We checked on the well-studied samples, like $Bi_2Sr_2CaCu_2O_8$, to make sure that our ARPES system works in a normal condition. In particular, we find that the measured data show similarity to the electron-doped $(Nd,Ce)_2CuO_4$ data[2] at relatively high doping. These observations indicate that the measured data are intrinsic to the measured sample; they are not from experimental artifacts.

Since photoemission measures only the occupied state because of the Fermi cutoff, the observation of states well above the "Fermi level" of the pristine CCOC326 indicates that the real Fermi level for those measurements after Rb deposition lies at higher kinetic energy. At least it should be higher than that of the observed states.

The usual way of determining the sample Fermi level is no longer useful for the Rb-deposited CCOC326, we have to find another way of setting the Fermi level. We found that the overall electronic structure evolution

with electron doping in our present case is similar to that in electron-doped $(Nd,Ce)_2CuO_4$[2]. In $(Nd,Ce)_2CuO_4$ with different electron doping levels (0, 0.04, 0.10 and 0.15), the Fermi level was determined from the usual way of measuring the gold Fermi cutoff. Particularly, the states near the antinodal (π,0) point approach the Fermi level for the doped samples[2]. We also find that the antinodal states in Rb-deposited CCOC326 lie at higher kinetic energy than the nodal states, similar to the electron-doped $(Nd,Ce)_2CuO_4$[2]. These observations make it reasonable to set the highest energy cutoff of the antinodal measurements (Supplementary Fig. 3c) as the corresponding Fermi level, as marked by the white dashed lines in panels of Supplementary Fig. 3c. For the same set of three momentum cut measurements at each Rb deposition stage, they have the same Fermi level as that determined from the antinodal states; in this way, the Fermi level for all the measurements are determined, as shown in Supplementary Fig. 3. The band structures by taking the energy scale relative to the Fermi level can be obtained and are shown in Fig. 2. This procedure makes it possible to investigate the electronic structure evolution in lightly electron-doped CCOC326, particularly the dramatic electronic structure evolution within the critical doping range of 0$\sim$0.04 that was not accessed in previous ARPES measurements[2].

The observation in Supplementary Fig. 3 that electronic states are detected above the normal Fermi level from gold is very rare in ARPES measurements. This could be due to the special case that the measured insulating sample surface is deposited with Rb. With Rb deposition, the top layer(s) of the sample surface is doped with electrons. But the sample beneath the electron-doped layer(s) remains insulating as before. Therefore, it is possible

that there is a band-bending between the top electron-doped layer(s) and the underneath insulating layers that gives rise to the Fermi level difference between the two parts. Although the exact origin of the Fermi level difference needs further investigations, we note that the data in Supplementary Fig. 3 are intrinsic and highly reproducible. Since we are dealing with relatively large energy scale change with doping in the present work, the slight uncertainty in the Fermi level will not affect our main results and conclusions.

[1] Hu, C. *et al.* Evidence for multiple underlying Fermi surface and isotropic energy gap in the cuprate parent compound $Ca_2CuO_2Cl_2$. *Chin. Phys. Lett.* **35**, 067403 (2018).

[2] Armitage, N. P. *et al.* Doping dependence of an n-type cuprate superconductor investigated by angle-resolved photoemission spectroscopy. *Phys. Rev. Lett.* **88**, 257001 (2002).

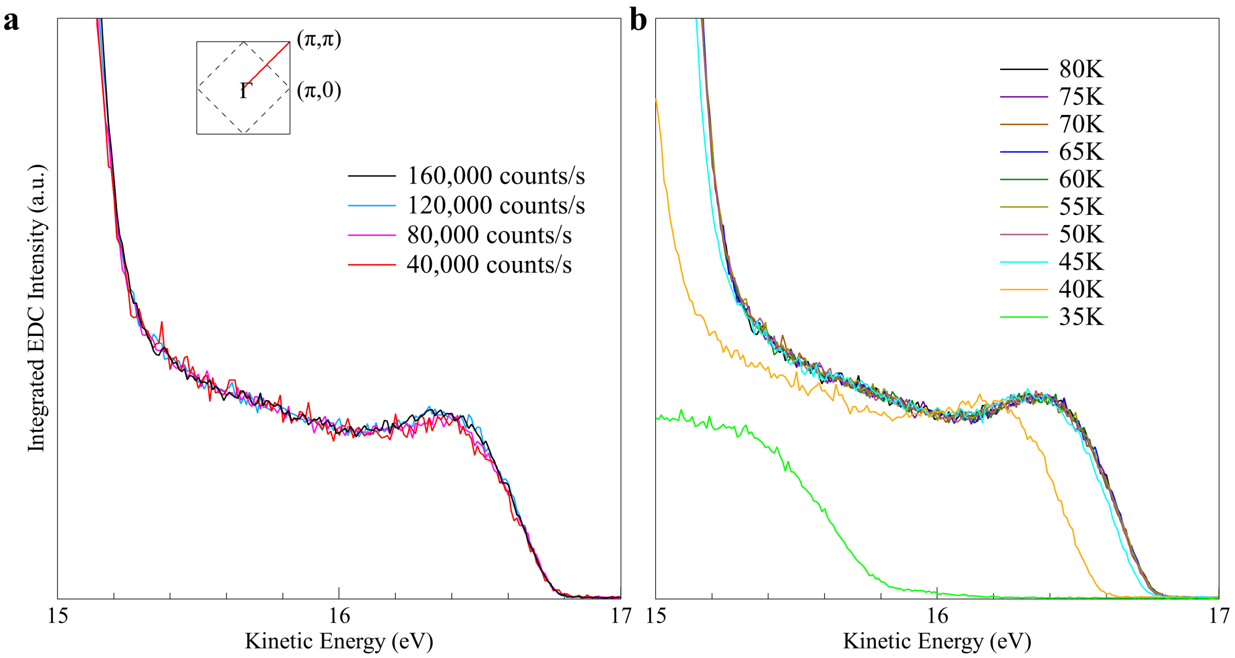

Supplementary Fig. 1. **The charging test of $Ca_3Cu_2O_4Cl_2$ sample with different photon flux and at different sample temperatures.** (a) Photoemission spectra (EDCs) taken on CCOC326 at 80 K with different photon flux. The EDCs are momentum-integrated spectra from the measurement along the nodal direction; the location of the momentum cut is marked as a red line in the inset. All the measured spectra show little change with the variation of the photon flux, which is proportional to the measured intensity as marked in the figure (counts/second). This indicates that little charging effect is present in measuring CCOC326 at 80 K. (b) EDCs taken on CCOC326 at different temperatures. The measured spectra show little change with temperature variation from 80 K down to 50 K. Obvious energy shift towards high-binding energy appears for the measurements at 40 K and becomes particularly serious for 35 K, signaling a strong charging effect due to the insulating nature of the CCOC326 parent compound. As a compromise, we chose a measurement temperature at ∼50 K to avoid sample charging problem on the one hand, and to have more efficient Rb absorption on the other hand.

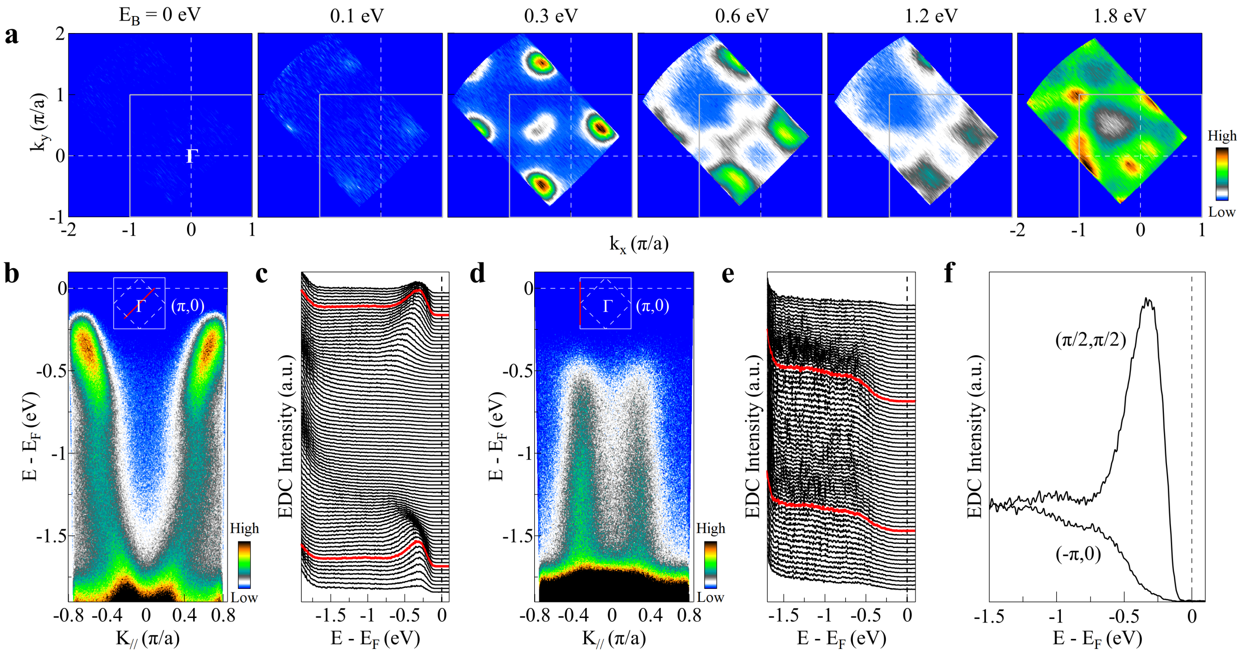

Supplementary Fig. 2. **Electronic structure of pristine $Ca_3Cu_2O_4Cl_2$.** (a) Constant energy contours of CCOC326 measured at 55 K, obtained by integrating the spectral weight over a 60 meV energy window at binding energies of 0, 0.1, 0.3, 0.6, 1.2 and 1.8 eV. (b) Band structure measured along the (-π,-π)-(π,π) nodal direction. The location of the momentum cut is shown as a red line in the inset. (c) Corresponding photoemission spectra (energy distribution curves, EDCs) of the band in (b). The red curves represent the EDCs that are closest in energy position to the Fermi level. (d,e) Same as (b,c) but measured along the (-π,-π)-(-π,π) zone face. The location of the momentum cut is shown in the inset of (d). (f) The EDCs taken at the ($\pi/2$,$\pi/2$) nodal point and the (-π,0) antinodal point.

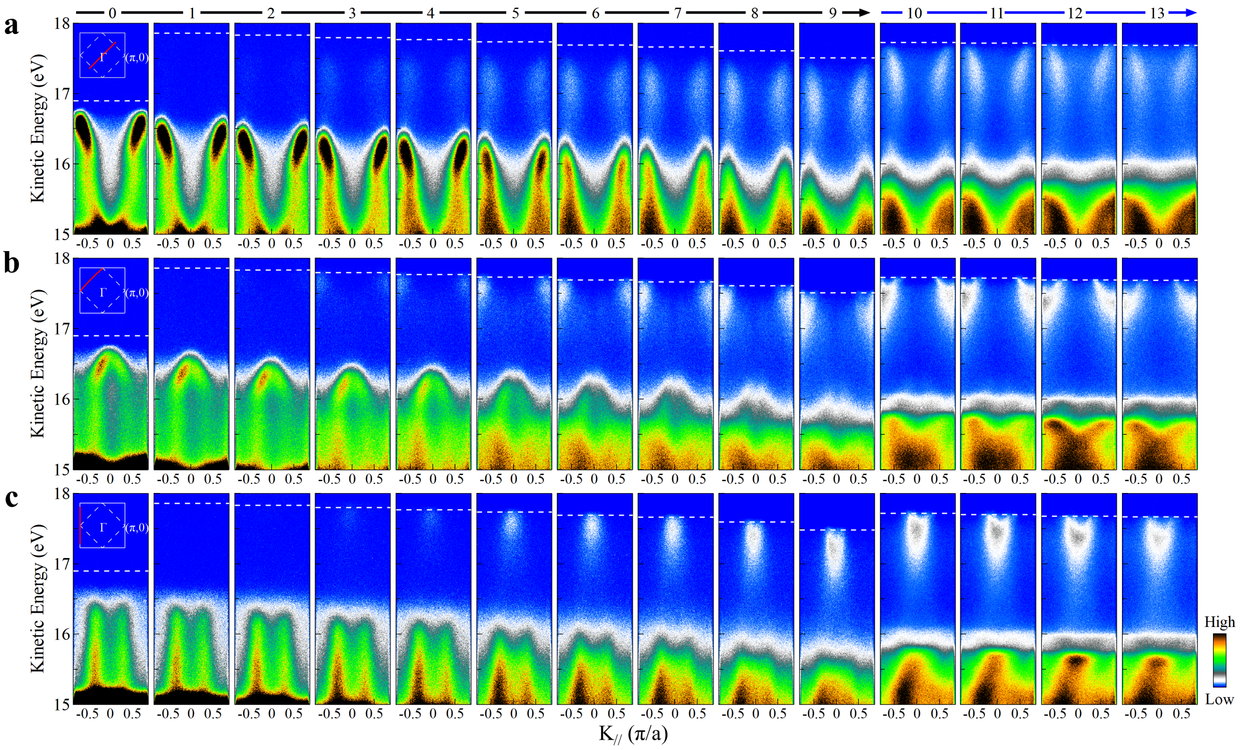

Supplementary Fig. 3. **Raw data of the band structure evolution of $Ca_3Cu_2O_4Cl_2$ with Rb deposition.** The energy scale used here is the measured photoelectron kinetic energy. After considering the Fermi level for each set of measurements, the same data are shown in Fig. 2 with an energy scale that is referenced to the Fermi level of the measured sample, E - E_F. (a) Doping evolution of band structure along the (-π/2,-π/2)-(π/2,π/2) nodal momentum cut. (b) Band structure evolution with Rb deposition for the (-π,0)-(0,π) momentum cut. (c) Band structure evolution with Rb deposition along the (-π,-π)-(-π,π) cut. The locations of the three momentum cuts are illustrated by the red solid line in the inset of panels (a), (b) and (c), respectively. The dashed white line in each panel represents the energy position of the Fermi level. The same set of data along three different momentum cuts after each Rb deposition stage have the same Fermi level.
